# Dimeticone 4% liquid gel found to kill all lice and eggs with a single 15 minute application

**DOI:** 10.1186/1756-0500-4-15

**Published:** 2011-01-25

**Authors:** Ian F Burgess, Nazma A Burgess

**Affiliations:** 1 Medical Entomology Centre, Insect Research & Development Limited, Stow-cum-Quy, Cambridge, UK

## Abstract

**Background:**

Dimeticone 4% lotion is an effective and widely accepted treatment for head louse infestation. However, it is a highly mobile fluid that some people find difficult to apply and is mainly left on the hair for 8 hours or overnight. User preference is for a more manageable and viscous product that can be used with a short application time.

**Findings:**

This proof of concept study in 41 people investigated dimeticone 4% liquid gel, a product that is easier to apply than the lotion, applied for 15 minutes on two occasions a week apart. We found that head lice were eliminated from all participants following the first application of product. We did not find lice of any stage on any participant during four post treatment assessments and particularly, unlike other treatments, no young nymphs on days 1 and 6 prior to the second treatment, indicating ovicidal as well as pediculicidal activity.

**Conclusions:**

Dimeticone 4% liquid gel has demonstrated efficacy greater than other similar products and the evidence obtained indicates elimination of head louse infestation with a single 15 minute application.

**Trial registration:**

Current Controlled Trials ISRCTN59227204

## Background

Dimeticone 4% lotion is a physically acting pediculicide based on 4% high molecular weight dimeticone in a cyclomethicone base. Since its introduction in 2006, dimeticone 4% lotion (Hedrin^® ^4% lotion) has become a market-leading product in several countries. Early studies showing equivalence with or superiority to insecticide products [1,2] were not received enthusiastically by some commentators because the cure rate of around 70% was considered less than optimum [3,4]. The rate of successful treatment in these studies was lower than expected at least partly due to rapid reinfestation of participants because relatively few individuals were treated in each community, many of which had a high prevalence of infestation. A more recent study involving whole communities in Turkey showed improved efficacy above 90% [5].

All previous investigations, and consumer feedback, have noted the fluid mobility of dimeticone 4% lotion and a readiness of the product to drip from some hair types. It is possible that flow of the lotion away from the roots of the hair may have influenced the treatment outcome on some trial participants if insufficient product remained in contact with lice and especially louse eggs. In response to discussions of this problem a more viscous preparation, Hedrin^® ^4% liquid gel, was developed by the manufacturer (Thornton & Ross Ltd, UK). The new liquid gel product was initially evaluated by our group in a proof of concept study using a 1 hour application time [IFB, NAB, ER Brunton, unpublished data]. In this study, we found the liquid gel eliminated infestation from more than 95% of the participants. In response to further feedback from consumers wishing for a shorter application time, this study was designed to determine whether the product is as effective using a reduced application time of 15 minutes.

### Study design

This was a single-centre, non-randomised, single-arm proof of concept study conducted in conformity with the principles of Good Clinical Practice and the Declaration of Helsinki. Ethical approval was granted by Sheffield Research Ethics Committee, reference 10/H1308/7.

All procedures were performed during visits to participants at home using essentially the same methods described in previous studies [1,2]. We confirmed that participants had an active head louse infestation by detection combing prior to treatment on day 0. We did not count lice or louse eggs on each person at the time of enrolment but measured the level of infestation by the rapidity of finding lice during combing. Participants confirmed they had not been treated with pediculicides for 2 weeks or with antibiotics or hair dyes for 4 weeks, were asked about possible allergies or sensitivities to treatment, and provided informed consent prior to entry. We assessed for possible persistence of infestation using a plastic head louse detection comb (PDC, KSL Consulting, Denmark) during follow-up visits on days 1 and 6 between treatments. On each occasion, the person was combed systematically for several minutes with actual time dependent upon hair length and thickness. The protocol required a second treatment to be applied to all participants on day 7 irrespective of whether lice had been found, with further combing checks for lice using the PDC on days 11 and 14 to confirm outcome of treatment.

### Study results

The study enrolled 41 participants from 19 families, with ages ranging from 2 - 44 (median 10 years), between 4^th ^March and 17^th ^May 2010. Of these 30 (73%) were female. The age structure of this cohort was non-significantly different from previous studies, but did have a smaller proportion (7/41, 17.1%) in the 6-9 years age group compared with 1-5 years (10/41, 24.2%) and 10-15 years (16/41, 39.0%) than in most previous studies. Other demographic characteristics such as hair length, thickness, degree of curl, and dryness/greasiness were similar to groups observed in previous studies. The level of infestation (heavy infestation = >1 louse with one stroke of the comb; moderate infestation = 1 louse with one stroke of the comb; light infestation = first louse found after several strokes of the comb), was significantly (p = 0.0295) higher with respect to heavy and moderate infestations than found in the previous investigation of liquid gel (ISRCTN50373146), using a 1 hour application of product. Using these criteria, we found eight cases of heavy infestation, 13 of moderate infestation, and the remainder were light infestations. However, in most cases, the actual intensity of infestation was greater than indicated by this semi-subjective measure and all participants had more than 10 viable eggs in their hair. There was no identifiable relationship between intensity of infestation and hair length or thickness. Most participants either reported seeing lice in rinsing water while washing out the treatment or found the dead bodies of lice on the pillow or during grooming the morning following treatment.

Follow up detection combing of participants is designed to not only measure the initial therapeutic impact of treatment on the infestation but also to look for newly emerged nymphs, which would have emerged from eggs not killed by the treatment. Detection combing of participants after treatment in this study was unable to find live lice of any stage on anyone following the first application of dimeticone 4% liquid gel. Unlike all previous studies (Table 1), no nymphal lice were found during the week following treatment, indicating that all louse eggs were prevented from hatching by the first treatment application.

**Table 1:** Comparison of reported numbers of participants found with lice and with louse nymphs following first treatment using different dimeticone based products

Study	Lice present before day 7	Percent	Nymphs present before day 7	Percent	Probability
Hedrin lotion overnight [1] *	91/127	71.6	86/127	67.7	a
ISRCTN47755726 Hedrin lotion overnight [2] *	32/43	74.4	27/43	62.8	a
ISRCTN15117709 Nyda L lotion overnight [6] **	26/73	35.6	Data not available	-	b
ISRCTN50373146 Hedrin liquid gel 1 hour *	3/42	7.1	2/42	4.8	c
ISRCTN59227204 Hedrin liquid gel 15 min	0/41	0.0	0/41	0.0	c

* Treatments used with two applications 7 days apart

** Treatment instructions originally recommended two applications with combing but now indicate one application may be effective with a repeat after 8 days if lice persist

Probability: Differences between groups were determined using Fisher's exact test. Groups with the same letter were not significantly different from each other. Differences were detected between groups 'a' and 'b' (p < 0.001) and groups 'b' and 'c' (p < 0.001)

There were no adverse events related to treatment and no serious adverse events. The 8 reported adverse events in five people included 3 lower limb injuries (2 bruised heels from poorly supported trainer shoes, one broken tibia after falling from a trampoline), one case of hand injuries from using crutches, a prescheduled overnight stay in hospital followed by a photocoagulation operation for endometriosis, and 2 incidents of listlessness and loss of appetite after returning from school, both of which passed within a few hours.

The only apparent drawback for this treatment regimen was that after treatment 35/41 (85.4%) participants found the product more difficult to wash out than some other preparations and 32 (70.1%) found their hair felt more oily than normal. However, all the 38 who had had prior experience of using dimeticone-based preparations expressed a preference for using the liquid gel, based on its level of efficacy and the convenient application time.

## Discussion

We have found that Hedrin 4% dimeticone liquid gel applied for 15 minutes not only eliminated all cases of head louse infestation, using the standard approach to treatment of two applications a week apart, but also eliminated all mobile stages and inhibited any emergence of louse nymphs from eggs following the first treatment.

Various silicone formulations for control of head lice are now available, mainly on European markets. Two widely used dimeticone based preparations, Hedrin^® ^4% lotion (Thornton & Ross Ltd, Huddersfield, UK) and Nyda^® ^L (G. Pohl-Boskamp GmbH & Co. KG, Hohenlockstedt, Germany) both employ an 8 hour or overnight application but neither have shown either complete eradication of infestation or complete inhibition of louse egg hatching in the clinical evaluations conducted so far [1,2,5,6]. Both these preparations are designed to evaporate during the treatment process, which means that the majority of the fluid applied is the less active solvent material in the product. Consequently, although the hair may appear to be saturated it may not be adequately coated with product, which reduces the likelihood of killing louse eggs. In contrast, the liquid gel employs a non-volatile vehicle based on a polyethylene glycol/polypropylene glycol dimeticone co-polymer with silica silylate thickener, plus 1,6,10-dodecatrien-3-ol,3,7,11-trimethyl as a surface tension modifier. None of these components has any specific activity against lice other than to reduce flow of the material along hair shafts and to facilitate adhesion of the dimeticone to the louse or louse egg surface.

In therapeutic terms, killing lice is relatively easy and the introduction of physically acting materials such as dimeticone has greatly facilitated elimination of both adult and nymphal lice from both individuals and the community. However, consistent prevention of egg hatch was always a difficult goal to achieve even before the evolution of resistance to neurotoxic insecticides because most insecticide based products were not ovicidal. A few were able to penetrate the chorionic membrane through use of monoterpene components [7,8] and some others, although unable to kill the embryos, were able to prevent nymphs from hatching or surviving through deposition of residual insecticide on eggs and hairs that killed the insects after emergence [9]. Nevertheless, no product in the past could be considered reliably effective to kill all lice and eggs with a single application.

In the case of the liquid gel, unlike some other dimeticone based lotions, there is no volatile component so louse eggs should be thoroughly coated during the treatment process. Also, because some components of the product were found to be more difficult to wash out, it appears a residue of active material remains on the hairs, lice, and louse eggs even after most of the product has been removed with shampoo. We believe this coating occludes the respiratory structures of the insects and their eggs long past the actual treatment phase. In the case of lice, the physical occlusion due to high molecular weight dimeticone has been shown to disrupt water management [10]. However, louse eggs need to retain water rather than eliminate it. Prolonged contact with silicone would permit the low surface tension material to flow throughout the aeropyle structure and allowing contact between the silicone and the chorionic membrane. *In vitro *studies have indicated that this is facilitated by the 1,6,10-dodecatrien-3-ol,3,7,11-trimethyl (Penetrol™) component of the liquid gel but this component did not appear to contribute to treatment outcome for the lotion formulation in one clinical study [5]. Prolonged contact of silicone with the egg would result in two effects. First, the physical plug of dimeticone in the complex capillary tubes of the aeropyle structure would limit gaseous exchange, despite the relative gas permeability of siloxanes. Additionally there could be some lipid disruption of the chorionic membrane so that it would be less protective of the louse embryo, especially as it nears the point of hatching.

This study has shown an improved outcome of treatment using dimeticone 4% liquid gel than any previous study of a siloxane based product. Effective use of the product in communities where previous investigations using other treatments have been less effective gives confidence that our results are likely to be generalisable to a wider community, and feedback from study participants and other contacts using the product suggests this is the case. The result is encouraging and indicates that coordinated treatments using Hedrin^® ^4% liquid gel, in a similar manner to the way we used dimeticone 4% lotion in our study in Turkey [5], could reduce the burden of louse infestation in communities more effectively than using less predictably effective lotion preparations.

## Competing interests

IFB has been a consultant to Thornton & Ross Ltd and to various other makers of pharmaceutical products, alternative therapies, and combs for treating louse infestations. NAB is not aware of any competing interests.

## Authors' contributions

Conceived, designed, and obtained approvals for the study: IFB, with input from NAB. Performed the clinical interventions: IFB. Followed up participants: NAB. Wrote the paper: IFB, with comments from NAB. Both authors have read and approved the final version of the manuscript.
